# Histone Deacetylase Inhibitors and Diabetic Kidney Disease

**DOI:** 10.3390/ijms19092630

**Published:** 2018-09-05

**Authors:** Mitchell J. Hadden, Andrew Advani

**Affiliations:** Keenan Research Centre for Biomedical Science and Li Ka Shing Knowledge Institute of St. Michael’s Hospital, Toronto, ON M5B 1W8, Canada; haddenm@smh.ca

**Keywords:** histone, epigenetics, kidney disease, diabetes, nephropathy, acetylation

## Abstract

Despite recent clinical trial advances and improvements in clinical care, kidney disease due to diabetes remains the most common cause of chronic kidney failure worldwide. In the search for new treatments, recent attentions have turned to drug repurposing opportunities, including study of the histone deacetylase (HDAC) inhibitor class of agents. HDACs are a group of enzymes that remove functional acetyl groups from histone and non-histone proteins and they can affect cellular function through both epigenetic and non-epigenetic means. Over the past decade, several HDAC inhibitors have been adopted into clinical practice, primarily for the treatment of hematological malignancy, whereas other existing therapies (for instance valproate) have been found to have HDAC inhibitory effects. Here we review the current HDAC inhibitors in the clinic and under development; the literature evidence supporting the renoprotective effects of HDAC inhibitors in experimental diabetic kidney disease; and the adverse effect profiles that may prevent existing therapies from entering the clinic for this indication. Whereas recent research efforts have shed light on the fundamental actions of HDACs in the diabetic kidney, whether these efforts will translate into novel therapies for patients will require more specific and better-tolerated therapies.

## 1. Introduction

Kidney disease due to diabetes is the most common cause of chronic kidney failure across the globe [1]. When kidney disease progresses to the point that the kidneys are no longer able to meet the eliminatory requirements of the individual, end-stage kidney disease (ESKD) occurs, demanding treatment with renal replacement therapy by either dialysis or kidney transplantation. Over the years, advances in therapeutics, structures of care, and overall population health have reduced the incidence of ESKD among people with diabetes [2]. However, as a consequence of the global pandemic of diabetes [3], the absolute number of individuals with diabetes and ESKD continues to climb [2]. In a search for new therapies to stem the tide of this potentially catastrophic long-term complication, researchers have explored various drug repurposing opportunities [4], amongst them the histone deacetylase (HDAC) inhibitor class of agents. During the course of the past decade, a number of preclinical studies have emerged (including those from our own group [5,6]) that have demonstrated the efficacy of various HDAC inhibitors in experimental models of diabetic kidney disease. Whereas these findings have shed new light on the pathogenetic mechanisms that cause kidney disease in diabetes, it remains to be determined whether HDAC inhibitors will find a clinical niche for the treatment of complex chronic diseases such as diabetic kidney disease. Here, we spotlight the current need for new treatments for diabetic kidney disease, we briefly summarize the biological effects of HDACs and the various classes of pharmacological HDAC inhibitor that have been developed to date and we review the preclinical studies that have reported the effects of HDAC inhibitor treatment in experimental diabetic kidney disease.

## 2. A Note on Terminology that Reflects the Changing Face of Diabetic Kidney Disease

The historical textbook definition of kidney disease in diabetes will refer to the condition as “diabetic nephropathy” which, along with “diabetic retinopathy” and “diabetic neuropathy” is one of the three classical microvascular complications of diabetes. The term “diabetic nephropathy” denotes a chronic condition, originally described in the 1980s [7], that is characterized by the progressive appearance of incipient and then overt albuminuria that precedes a decline in glomerular filtration rate (GFR), culminating in ESKD. This clinical condition is often associated with specific histopathological features, most notably the classical Kimmelsteil-Wilson lesion of nodular glomerulosclerosis. In the decades that have followed the original characterization of diabetic nephropathy, it has become apparent that many individuals with diabetes develop clinically significant kidney disease that does not follow the progressive pattern originally described. Albuminuria may not progress and it may regress [8], many individuals develop a decline in GFR in the absence of albuminuria [9] and many individuals with diabetes can develop kidney failure in the absence of histopathological features of classical “diabetic nephropathy” [10]. As a result, recent years have witnessed a shift in preference for the term “diabetic kidney disease” over “diabetic nephropathy”, to reflect the multifarious nature of kidney disease occurring in a person with diabetes. For patient populations, the term “diabetes and chronic kidney disease (CKD)” probably better reflects the pathogenesis of the condition, given that many people with diabetes have comorbidities such as hypertension, atherosclerosis or age-related changes which may also contribute to kidney disease development [11]. Here, however, we focus on pre-clinical experimentation in which the effects of HDAC inhibitors have been studied in rodent models of diabetes, without other comorbidity, and we have thus chosen to employ the term “diabetic kidney disease”.

## 3. Diabetic Kidney Disease: Scope of the Problem

Diabetes affects over 451 million people worldwide [12] and diabetic kidney disease is the most common cause of CKD, accounting for approximately 40% of all cases [13]. When a person with diabetes develops kidney disease that progresses to ESKD, that person has a particularly poor prognosis. For instance, data from the United States (U.S.) Renal Data Systems report show that the five-year survival rate for people with diabetes and ESKD is only approximately 38% [14]. Most people with diabetes and kidney disease, however, will never reach the stage of requiring treatment with dialysis or kidney transplantation, but will instead die from cardiovascular disease [15]. In an Italian population of 15,773 individuals with diabetes and both albuminuria and a reduced estimated GFR (eGFR), for example, the crude mortality rate was 90.35 events per 1000 patient years (95% confidence interval (C.I.), 83.41–97.08) compared to a crude mortality rate of 19.87 (95% C.I., 18.87–20.86) for individuals with diabetes and without either albuminuria or a reduced eGFR [16]. This burden of morbidity and mortality comes at substantial cost to the health care system. In 2015, total Medicare spending on CKD and ESKD was almost $100 billion [14].

Current treatments for diabetic kidney disease are aimed at slowing the progression of renal decline and include control of blood glucose levels and control of blood pressure, particularly in the latter case, with agents that block the renin angiotensin system (RAS) [17,18,19,20]. More recently, evidence has begun to emerge supporting a renoprotective benefit of sodium-glucose cotransporter 2 (SGLT2) inhibitor therapy and this, together with RAS-blockade, probably represents current best care for most patients with Type 2 diabetes and kidney disease. SGLT2 inhibitors are a class of oral anti-hyperglycemic agents that lower blood glucose levels by preventing reabsorption of glucose from the urinary filtrate by the proximal tubule of the kidney [21]. In 2015 and 2016, the results of the EMPA-REG OUTCOME study surprised many in the diabetes, cardiovascular, and nephrology clinical care and research communities by demonstrating the benefits of SGLT2 inhibition on both cardiovascular and renal outcomes [22,23]. The EMPA-REG OUTCOME study examined the effects of the SGLT2 inhibitor, empagliflozin on cardiovascular mortality and morbidity in individuals with Type 2 diabetes at high cardiovascular risk and reported a 14% relative risk reduction in the primary endpoint (death from cardiovascular causes, non-fatal myocardial infarction, or non-fatal stroke) with empagliflozin compared to placebo [23]. In that study, empagliflozin treatment was also associated with a 39% relative risk reduction in renal endpoints (defined as progression of albuminuria (urinary albumin:creatine ratio >300 mg/g; doubling of serum creatinine and an eGFR <45 mL/min/1.73 m^2^; initiation of renal replacement therapy; or death from renal disease) (hazard ratio 0.61; 95% C.I. 0.53–0.70, *p* < 0.001) [22]. Despite recent successes, such as this however, renal decline still continues in many individuals with diabetes (incident or worsening nephropathy occurred in 12.7% of individuals in the empagliflozin-treatment arm of EMPA-REG OUTCOME [22]) and new treatments are needed. In the search for new treatments, considerable interest has arisen in drug repurposing (or repositioning) opportunities. Drug repurposing is an attractive approach that accelerates and de-risks research and development by employing therapies with proven bioavailability and safety, reducing cost and expediting clinical use [24]. Among the various repurposing opportunities that have been explored over the past decade have been the HDAC inhibitor class of agents that have been developed primarily for the purpose of treating hematological malignancy.

## 4. Histone Deacetylases and Their Biological Effects

**Figure 1:**
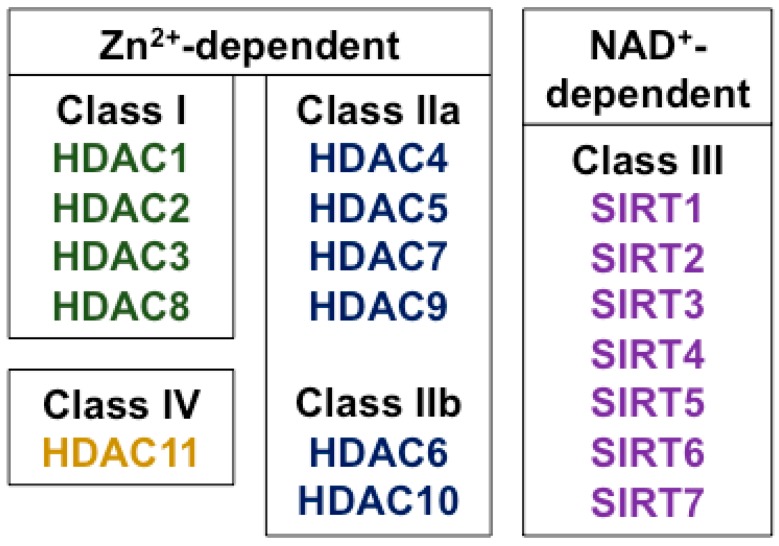
Classes of histone deacetylase (HDAC) enzymes in humans.

HDAC enzymes catalyze the removal of acetyl groups from ε-amino-acetylated lysine residues on both histone and non-histone proteins. There are at least 18 different HDAC enzymes and they are categorized into four classes according to their homology to yeast HDACs, in which they were first discovered [25] (Figure 1). The “classical family” of HDACs (Classes I, II, and IV) require Zn^2+^ for their enzymatic effects. Class I HDACs include HDAC isoforms HDAC1, 2, 3, and 8; Class II HDACs are subdivided into Class IIa (HDACs 4, 5, 7, and 9) and Class IIb (HDACs 6 and 10). Class III HDACs do not require Zn^2+^, but use NAD^+^ as a co-factor. They are known as the sirtuins and, in mammals, they comprise at least seven members (SIRTs 1-7). Because of their different mechanism of action, the sirtuins are not inhibited by known Class I and Class II HDAC inhibitors [26]. HDAC11 shares some homology with both Class I and Class II HDACs and is the sole member of the fourth class of HDACs (Class IV).

**Figure 2:**
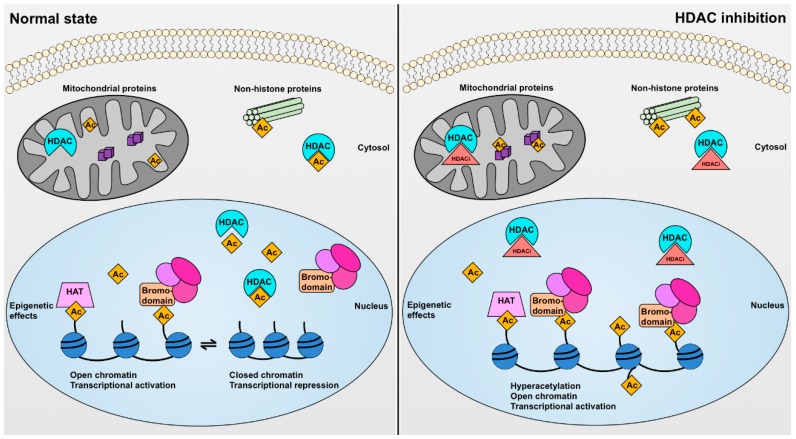
Cellular effects of histone deacetylases (HDACs) and HDAC inhibitors (HDACis). In the normal state (left) HDAC enzymes mediate the enzymatic removal of functional acetyl groups (Ac) from lysine residues on histone and non-histone proteins. In the nucleus, histone protein acetylation (regulated by histone acetyltransferase (HAT) enzymes) can epigenetically affect gene transcription by causing a more open chromatin configuration permitting access by the transcriptional machinery and by serving as a recognition point for bromodomains possessed by transcriptional regulatory complexes, whereas histone deacetylation has the opposite effect. Some HDAC enzymes also have effects on non-histone proteins. For instance, SIRT3, SIRT4, and SIRT5 are mitochondrial proteins, whereas HDAC6 is localized primarily in the cytosol where it deacetylates proteins including α-tubulin. Pharmacological HDAC inhibition (right) can increase the acetylation of both histone and non-histone proteins resulting in both epigenetic and non-epigenetic effects on cell function.

There are several different ways through which HDACs may exert their biological effects. These can be considered as epigenetic effects (which occur in the nucleus) and non-epigenetic effects (which occur in the cytosol or mitochondria) (Figure 2). HDACs can epigenetically affect gene transcription by the post-translational modification (specifically deacetylation) of histone proteins. Histones are the protein spools around which DNA coils to form the nucleosome core particle, the fundamental unit of chromatin, and their acetylation or deacetylation can affect gene transcription in at least two different ways. Firstly, when lysine (or arginine) residues of histone tails are unmodified they possess a positive charge. This enables the histone proteins to interact more closely with the negatively charged sugar-phosphate DNA backbone resulting in chromatin compaction, limiting accessibility by the transcriptional machinery and reducing gene transcription. Conversely, acetylation of lysine residues on histone tails neutralizes the positive charge causing a more open conformation that facilitates gene transcription, at least in theory. This may occur, for instance, when HDAC enzymes are pharmacologically inhibited (Figure 2). Secondly, histone acetylation can facilitate the recruitment and assembly of transcriptional regulatory complexes by forming a recognition point for bromodomains [27,28], which are modules of approximately 110 amino acids commonly present in proteins that interact with chromatin [29,30,31] (Figure 2). In the nucleus, HDAC isoforms rarely function in isolation, rather most form large complexes with transcriptional co-repressors (e.g., NuRD-Mi2 complexes and core complexes containing nuclear receptor corepressor (*N*-CoR) or silencing mediator for retinoid and thyroid receptors (SMRT) [32]). 

Despite their name, HDACs do not solely deacetylate histone proteins. Indeed, some HDACs (e.g., HDAC6) exist almost exclusively in the cytoplasm [33]. Numerous non-histone proteins have been observed to undergo acetylation and deacetylation. For instance, one study identified 1750 acetylated proteins in MV4-11 cells [34]. It has been speculated upon that protein acetylation could rival protein phosphorylation in the extent of its biological effects [35]. However, the actual consequences of the acetylation or deacetylation of many cytosolic proteins are still largely undetermined. It is also possible for some HDACs to have deacetylase-independent effects. For example, in addition to its two catalytic domains, HDAC6 also possess a ZnF-UBP domain which enables it to bind to ubiquitinated proteins and a dynein motor-binding domain which enables it to bind to dynein and these protein-interacting partnerships enable HDAC6 to function in the disposal of misfolded proteins [33]. Such deacetylase-independent actions may be unaffected by the binding of small molecule inhibitors to the catalytic site of the protein. Finally, histone lysine residues can also be modified by other acyl groups, which may also be substrates for HDAC enzymes, HDAC3 for instance being able to decrotonylate histone proteins [36].

## 5. Pharmacological HDAC Inhibitors

There are five classes of HDAC inhibitor compounds, classified according to their chemical structure: hydroxamic acid derivates (e.g., trichostatin A, vorinostat, rocilinostat, belinostat, and panobinostat); short chain fatty (aliphatic) acids (e.g., sodium butyrate, and valproate); cyclic peptides (e.g., romidepsin); benzamides (e.g., mocetinostat, entinostat, and Tubastatin A); and sirtuin inhibitors (e.g., nicotinamide) (Table 1) [26,37,38,39]. Most HDAC inhibitors target the Zn^2+^ domain and thus commonly are effective in inhibiting multiple different HDAC isoforms, although are ineffective against the sirtuins. Because of their different mode of action, sirtuin inhibitors are not considered further in this review. Amongst the HDAC inhibitors that do target Zn^2+^-dependent isoforms, agents may have broad-spectrum effects (commonly called pan-HDAC inhibitors), class effects or isoform-specific effects. 

The hydroxamic acid derivates were the first HDAC inhibitor class to receive regulatory authority approval. These agents generally have poor isoform-specificity. Trichostatin A is one of the first hydroxamic acid HDAC inhibitors to have been discovered and it is not used clinically because of unfavorable absorption, distribution, metabolism, excretion, and toxicity (ADMET) properties. Vorinostat (also called suberoylanilide hydroxamic acid, SAHA) was identified as an HDAC inhibitor based upon its structural similarity to trichostatin A [40] and, like Trichostatin A, it inhibits Class I and II HDACs [40]. Vorinostat was the first HDAC inhibitor to be adopted into clinical practice, receiving U.S. Food and Drug Administration (FDA) approval for the treatment of cutaneous T cell lymphoma in 2006. As of 2018, four other HDAC inhibitors have received regulatory approval, all within the field of hematological malignancy (Table 1). Belinostat and panobinostat are both also hydroxamic acid derivates and broad-spectrum HDAC inhibitors, currently approved by the FDA for the treatment of peripheral T-cell lymphoma and multiple myeloma, respectively [41,42]. Romidepsin is a structurally unique cyclic peptide and inhibitor of Class I HDACs approved by the FDA for the treatment of cutaneous T cell lymphoma and other peripheral T cell lymphomas [43]. Chidamide is also a Class I selective HDAC inhibitor [44]. It belongs to the benzamide class and is approved in China for the treatment of peripheral T cell lymphomas [45]. A number of other HDAC inhibitors with varying degrees of selectivity are also currently undergoing clinical trial (Table 1).

In addition to purpose-developed HDAC inhibitors, several other pharmacological agents have been discovered to have HDAC inhibitory effects and are now undergoing investigation for this mode of action. Perhaps the best example is valproate. Valproate is a branched short chain fatty acid that is used in the treatment of epilepsy and bipolar disorder and in migraine prevention. Even though valproate has been used clinically since 1962 [46], its precise mechanisms of action have been unclear. However, in 2001, valproate was reported to have HDAC inhibitory effects (with greater selectivity for Class I HDACs over Class II HDACs) [47] and a number of studies have explored its actions in this context since. Other pharmacological agents used in clinical practice for different indications have also been reported to have HDAC inhibitory effects which may explain some of their pleitropic actions, as is the case for instance for the lipid-lowering agent, atorvastatin [48].

## 6. Evidence for Altered HDAC Activity in Diabetic Kidney Disease

The development of HDAC inhibitors for other indications and their successful adoption into clinical practice has encouraged exploration of their potential beneficial effects in diabetic kidney disease as a repurposing opportunity. Our group, and likely other investigators, became interested in exploring the possibility of repurposing HDAC inhibitors for the treatment of diabetic kidney disease because their integration into clinical care roughly coincided with the identification of epigenetic processes as important mediators of diabetes complications. More specifically, the long-term follow-up of participants of landmark clinical trials of glucose-lowering in diabetes revealed that individuals who were initially randomized to intensive glycemic control experienced a persistent reduction in the risk of diabetes complications (including kidney disease) for many years after the completion of the initial trials [49,50], a paradigm commonly referred to as “metabolic memory” [51]. Epigenetic processes have been mooted as possible mediators of the phenomenon of metabolic memory, providing a molecular means whereby a transient environmental exposure can have persistent effects on cellular function [52]. Amongst all of the epigenetic processes identified to date, histone deacetylation has revealed itself to be the most amenable to therapeutic manipulation and thus HDAC inhibitors have begun to be explored as treatments for diabetic kidney disease in experimental models.

The area in which HDAC inhibitors have been most extensively studied has been in the field of cancer. In comparison to non-cancerous cells, cancer cells exhibit a relative sensitivity to HDAC inhibitors where the small molecules induce cell cycle arrest, differentiation, and programmed cell death [53]. This mode of action is thus unlikely to provide a means for end-organ benefit in diabetic kidney disease. Accordingly, in considering the utility of HDAC inhibitors for the treatment of diabetic kidney disease, what evidence (if any) exists that HDAC activity is altered in this disease setting? One of the first studies to examine the role that HDACs play in diabetic kidney disease was published by Noh and co-workers in 2009 [54]. In that study, the investigators reported an increase in the activity of the HDAC2 isoform in the kidneys of rats with diabetes induced by streptozotocin (STZ, an alkylating agent that is toxic to the insulin-producing ß-cells of the pancreatic islets), in Type 2 diabetic db/db mice and in proximal tubule lineage NRK-52E cells exposed to the profibrotic cytokine, transforming growth factor-ß1 (TGF-ß1) [54]. In one of our own studies, published in 2011, we observed that the proportion of glomerular nuclei positively immunostaining for acetylated histone H3 was reduced in mice 18 weeks after STZ-induced diabetes [5]. Wang and co-workers, reported increased expression of HDAC2, HDAC4, and HDAC5 in the kidneys of STZ-diabetic rats and db/db mice and increased expression of HDAC4 and HDAC5 in the kidneys of humans with diabetic kidney disease, with an inverse correlation between estimated glomerular filtration rate (eGFR) and the expression of either HDAC2, HDAC4, or HDAC5 [55]. However, increased HDAC expression or HDAC activity or decreased protein acetylation is not necessarily a universal finding in diabetic kidney disease. For instance, Cai and co-workers reported increases in acetylation of lysine residue 9 (K9) on histone H3 (H3K9ac) in the kidneys of diabetic mice [56] (which could indicate either an increase in histone acetyltransferase (HAT) activity or a reduction in HDAC activity) and Kosanam and co-workers reported an overall increase in protein lysine acetylation in the kidneys of STZ-diabetic transgenic (mRen-2)27 rats [57]. It is worth noting, however, that an increase in HDAC expression or activity is not a prerequisite in order for HDAC inhibitors to confer therapeutic benefits. By analogy, RAS-blockers are standard of care for people with diabetic kidney disease, despite diabetes being a low renin state [58]. Indeed, whereas total angiotensin converting enzyme (ACE) levels may be reduced in diabetic kidneys a redistribution of ACE may contribute to increased local RAS activity [59]. If HDACs do contribute to the pathogenesis of kidney disease in diabetes, it is likely that their effects are more precise than global HDAC actions in whole kidneys, being isoform-specific, site-specific, epigenome-specific and/or protein-substrate specific. Thus, much work needs to be done to define the precise actions of HDAC isoforms in individual cell-types in the diabetic kidney. In the meantime, with the development of pharmacological HDAC inhibitors continuing apace, what evidence exists that these agents can alter the natural history of experimental diabetic kidney disease?

## 7. HDAC Inhibitor Effects in Experimental Diabetic Kidney Disease

The first evidence that HDAC inhibitors may have beneficial effects on the kidney came in 2003 when investigators reported that the pan-HDAC inhibitor, trichostatin A reduced proteinuria in a murine model of lupus nephritis [60]. Since then, several studies have explored the effects of specific HDAC isoforms on kidney disease development, particularly with an emphasis on the development of renal fibrosis. These studies have been summarized in several recent reviews [26,37,61,62]. Here, we focus on the actions of pharmacological HDAC inhibitors, as they have been examined in the context of experimental models of diabetic kidney disease (Table 2). To date, the actions of HDAC inhibitors in diabetic kidney disease have been reported for the hydroxamic acid and short chain fatty acid classes of agents.

## 8. Hydroxamic Acids

### 8.1. Trichostatin A

One of the first hints that HDAC inhibitors may show efficacy in diabetic kidney disease came with data presented in a review article published in 2007. In that study, investigators reported that the broad-spectrum HDAC inhibitor, trichostatin A attenuated the upregulation of both α-smooth muscle actin (α-SMA) and fibronectin and downregulation of E-cadherin induced by hydrogen peroxide (H_2_O_2_) in NRK-52E cells [68]. In 2009, the same authors followed this initial report up with a more extensive exposition of the effects of HDACs in NRK-52E cells and in the kidneys of diabetic rodents [54], a study already alluded to above in the section on HDAC activity in the diabetic kidney. In that study, the authors reported that daily injection of trichostatin A (500 µg/kg subcutaneously) over a four week period to diabetic rats, decreased proteinuria and prevented the upregulation of renal expression of fibronectin, collagen I and α-SMA and downregulation of renal expression of tubule E-cadherin [54]. Similarly, trichostatin A prevented TGF-ß1 induced matrix protein upregulation and E-cadherin downregulation in NRK-52E cells [54]. These effects could be mimicked by the Class I-selective HDAC inhibitor, SK-7041 [69] and by knockdown of the Class I HDAC isoform, HDAC2 [54]. 

In the original report by Noh et al., in 2009, HDAC2 activity was increased by either TGF-ß1 or H_2_O_2_ in NRK-52E cells, whereas the antioxidant N-acetylcysteine prevented TGF-ß1 induced HDAC2 activation [54], pointing to the relationship between oxidative stress and HDAC actions. Oxidative stress has long been linked to the development of kidney disease in diabetes [70]. It can occur through either an increase in generation of reactive oxygen species (ROS) [70] or a reduction in the enzymatic activity of antioxidant repair systems [71]. One of the key proteins involved in the latter process is a protein termed thioredoxin interacting protein (TxnIP), which functions to limit the effects of the antioxidant repair enzyme, thioredoxin [72]. Our work [73], and that of others [74], has found that expression of TxnIP in the diabetic kidney is epigenetically regulated. In human mesangial cells cultured under normal glucose conditions, trichostatin A increased TxnIP expression whereas it had no effect on the upregulation of TxnIP induced by high glucose [74]. In further work by the same authors, a similar pattern of change was observed for expression of the gene encoding the secreted glycoprotein, osteopontin by mesangial cells; trichostatin A increasing osteopontin expression under normal glucose conditions but having no effect on the upregulation induced by high glucose [56]. Together these observations serve to highlight both that the effects of HDAC inhibitors can be influenced by a high glucose milieu and that their effects may not be universally salutary, increased TxnIP and osteopontin expression plausibly contributing to the development of kidney disease [75,76].

### 8.2. Vorinostat

Our own work, on the effects of HDAC inhibitors in experimental diabetic kidney disease, focused on the actions of the hydroxamic acid, vorinostat [5,6]. One of the earliest renal changes that occurs in diabetes is kidney enlargement, evident in both human [77] and experimental diabetes [78,79], where it may predict future risk of renal decline [80,81]. Although the precise molecular processes underlying renal enlargement are still incompletely understood, there appears to be an important role for epidermal growth factor (EGF) and its ErbB family receptor tyrosine kinase, epidermal growth factor receptor (EGFR) [82,83]. Because HDAC inhibition with vorinostat had been shown to decrease EGFR expression in other cells [84] and because vorinostat had been adopted into clinical practice for other indications, we decided to explore the effects of this agent on renal growth in early experimental diabetes. We found that vorinostat downregulated EGFR expression and decreased proliferation in cultured NRK-52E cells, whereas treatment of STZ-diabetic rats with vorinostat (50 mg/kg) by daily gavage decreased tubule cell proliferation (after three days) and glomerular hypertrophy and renal enlargement (after four weeks) [6].

In a separate study, we treated STZ-diabetic mice with vorinostat (50 mg/kg/day) for 18 weeks, observing an attenuation in the development of albuminuria and a reduction in both glomerular collagen IV deposition and mesangial matrix accumulation [5]. We noticed that these improvements in kidney injury were associated with a reduction in oxidative stress and in exploring the mechanisms underlying this, we focused on the paradigm of endothelial nitric oxide synthase (eNOS) uncoupling [5]. The term eNOS uncoupling describes a set of particular molecular events that lead to enhanced superoxide production. In the setting of hyperglycemia, ROS may react with eNOS-derived nitric oxide (NO) to form peroxynitrite, which can in turn oxidize the eNOS cofactor tetrahydrobiopterin leading to the generation of superoxide in preference to NO [85,86]. We observed that vorinostat decreased eNOS expression in cultured human umbilical vein endothelial cells and in mouse kidneys, which led us to speculate that this partial (but incomplete) reduction in eNOS with HDAC inhibition served to limit eNOS uncoupling and oxidative stress in diabetes [5]. Supporting a causal role for eNOS downregulation in the renoprotective effects of vorinostat in diabetic mice, we observed that the HDAC inhibitor was ineffective in providing kidney protection in STZ-diabetic eNOS knockout (eNOS^−/−^) mice [5].

## 9. Short Chain Fatty Acids

The other major class of HDAC inhibitor that has been studied in the context of diabetic kidney disease is the short chain fatty acid class, studies reporting the effects of both valproate and sodium butyrate.

### 9.1. Valproate

As already described, valproate (valproic acid or sodium valproate) has been employed clinically for the treatment of epilepsy, migraine, and bipolar disorders for many years, whereas it was more recently reported to be an inhibitor of Class I and Class II HDACs [47,87]. Khan and co-workers published two reports in 2015 in which they reported the effects valproic acid when administered to STZ-diabetic rats for eight weeks (150 mg/kg/day and 300 mg/kg/day orally) [63,64]. They found that valproic acid significantly attenuated tubule cell injury and renal fibrosis [63], as well as proteinuria development [64], accompanied by a reduction in the diabetes-associated upregulation of the pro-inflammatory transcription factor, nuclear factor kappa-light-chain-enhancer of activated B cells (NF-κB) [64]. Furthermore, the authors reported that valproic acid prevented the reduction in autophagy that they observed in the kidneys of rats after eight weeks of diabetes [64]. This observation would be aligned with a separate report that the HDAC4 isoform contributes to kidney injury in diabetes by inhibiting podocyte autophagy in a signal transducer and activator of transcription 1 (STAT1) dependent manner [55]. Also linking HDAC isoform inhibition to enhanced autophagy and renoprotection, albeit in a non-diabetic context, we recently reported that inhibition of HDAC6 attenuated proteinuria in a rat remnant kidney model and that HDAC6 inhibition enhanced autophagy by acetylating the master regulator of autophagy, transcription factor EB (TFEB) [88,89,90].

The renoprotective effects of valproate were also reported by a separate group of investigators in 2016 [65]. These investigators performed a late intervention study, inducing diabetes with STZ in rats and feeding the animals a high fat diet for 22 weeks before initiating treatment with valproate (200 mg/kg by daily oral gavage) for a further six weeks [65]. The authors reported that valproate treatment decreased proteinuria and glomerular matrix accumulation [65]. These functional and structural improvements were accompanied by molecular changes indicative of diminished endoplasmic reticulum stress and programmed cell death [65].

### 9.2. Sodium Butyrate

Two articles have reported the effects of HDAC inhibition with sodium butyrate in diabetic rats. The first was, again, conducted by Khan and co-workers [66] and performed in STZ-diabetic rats. Rats were treated with sodium butyrate (500 mg/kg) by daily intraperitoneal injection for either 21 days before diabetes induction or 21 days after diabetes induction [66]. In that study, treatment with sodium butyrate after diabetes induction lowered plasma glucose levels, whereas treatment prior to diabetes induction did not [66]. As was seen with valproate, treatment of rats with sodium butyrate after diabetes induction also lowered NF-κB expression and attenuated kidney injury and indices of matrix deposition [66]. Interestingly, as we had observed with vorinostat-treatment of diabetic mice, treatment of diabetic rats with sodium butyrate also lowered renal eNOS (and inducible nitric oxide synthase, iNOS) expression [66]. In terms of the mechanism behind the renoprotective effects of sodium butyrate, a recent study has pointed to an important role for the activation of nuclear factor erythroid 2-related factor 2 (Nrf2) [67]. Nrf2 is a basic leucine zipper protein that controls the expression of a number of antioxidant genes [91] and is known to be activated by sodium butyrate [92,93]. Dong and co-workers treated STZ-diabetic wildtype and Nrf2^−/−^ mice with sodium butyrate (5 g/kg/day in chow) for 20 weeks and observed that sodium butyrate attenuated diabetes-associated oxidative damage, inflammation, programmed cell death, fibrosis, and albuminuria in wildtype mice, but not Nrf2^−/−^ animals [67]. Furthermore, Nrf2 expression was observed to be diminished in the kidneys of diabetic wildtype mice and this diminution was prevented by sodium butyrate, prompting the investigators to conclude that sodium butyrate regulates Nrf2 at the level of transcription [67].

Finally, aside from the effects of previously recognized HDAC inhibitors, it is plausible that some of the actions of other agents may be mediated by their interference with HDAC activity. For instance, the renoprotective actions of the 3-hydroxy-3-methyl-glutaryl-coenzyme A reductase (HMG-CoA reductase) inhibitor, atorvastatin have been attributed to HDAC inhibiting effects of the statin [48].

## 10. Future Directions

As reviewed above, over the past decade a small body of literature has emerged purporting the renoprotective benefits of broad-spectrum HDAC inhibitors in diabetic rats and mice. All of the studies reporting the effects of HDAC inhibitors in experimental diabetic kidney disease to date have employed the STZ-model, which most likely reflects the comparative simplicity of diabetes induction with this approach. However, in the absence of studies employing STZ-independent models of diabetes caution should be exercised before extrapolating these findings more broadly. The reported benefits of HDAC inhibition largely relate to early renal changes of diabetes including nephromegaly, proteinuria and the overelaboration of profibrotic factors and matrix proteins. This may be a consequence of the fact that most rodent models of diabetes do not develop late-stage fibrotic changes that are typically associated with GFR decline [94]. That being said, broad-spectrum, class-specific or isoform-specific HDAC inhibitors have been reported to have anti-fibrotic effects in non-diabetic rodent models of renal fibrosis, including the unilateral ureteral obstruction model [95,96,97,98,99,100,101,102] and the rat remnant kidney model [88]. Given that common pathogenetic mechanisms are often shared during the development of renal fibrosis, regardless of the etiology, it could be postulated that HDAC inhibition may confer similar benefits on later stage renal decline. Similarly, the actions of HDACs in diabetes are not limited to those on the kidney and HDAC inhibitors may offer other metabolic benefits in the treatment of diabetes [103,104,105]. For instance, Class I HDAC inhibition was reported to enhance oxidative metabolism in skeletal muscle and adipose tissue, improving insulin sensitivity in db/db mice [106]; and inhibition of HDAC3 decreased pancreatic ß-cell programmed cell death and increased ß-cell proliferation, preventing diabetes onset in nonobese diabetic mice [107]. 

Even if HDAC inhibitors do provide clinical benefit for the treatment of diabetic kidney disease, however, this would be far from the sole requirement for a therapy to be adopted into clinical practice. Not least, novel therapies must exhibit a safety and tolerability profile that render them suitable for the long-term treatment of complex chronic diseases. In this respect, tolerability may be where the currently available HDAC inhibitors would likely fall short for this indication even if their efficacy were proven. Table 3 lists HDAC inhibitors currently approved by the U.S. FDA along with their common adverse effects and serious adverse effects. Whereas these adverse effect profiles may be deemed acceptable for the treatment of malignancy, a lower threshold of acceptability exists for the treatment of complex chronic diseases such as diabetic kidney disease. As an additional approach to the development of better-tolerated HDAC inhibitors, exploration of the HDAC inhibitory effects of agents originally employed for other indications warrants further attention. In this regard, valproate has been employed clinically for decades and is already used as a treatment for chronic conditions (seizures, migraine, and bipolar disorder). Many people treated with valproate will also have comorbid diabetes and many people receiving valproate will have received treatment whilst also taking other agents used to prevent the progression of kidney disease (e.g., RAS blockers). Furthermore, valproate may occasionally be used as a treatment for painful neuropathy in people with diabetes [108]. Thus, there is ample clinical experience of the use of this agent in people with diabetes, alone and in combination with other reno-protective therapies. Before launching into costly clinical trials of HDAC inhibition in patients with diabetic kidney disease, it should be possible to interrogate large population databases to search for clues as to whether kidney disease development or progression is altered in people with diabetes receiving valproate in comparison to people with diabetes receiving other therapies for seizures or neuropathic pain. 

Beyond tolerability, given the breadth of action of HDACs, isoform-selectivity is likely to be an important determinant of the success of HDAC inhibition if it is to be adopted into clinical practice for the treatment of diabetic kidney disease. Some class-specific HDAC inhibitors are currently undergoing clinical trial evaluation (e.g., mocetinostat and entinostat) (Table 1) [109,110], whereas few isoform-specific agents have been successfully developed to date [111]. Here, it is pertinent to note that HDAC isoforms (e.g., HDAC1 and HDAC2) often exhibit functional redundancy [112,113,114,115] and thus inhibition of some HDAC isoforms in isolation may prove to be clinically ineffective. Finally, as is almost invariably the case in the study of other agents considered for repurposing, the initial studies performed in preclinical models have explored HDAC inhibition as monotherapy. However, in clinical use, any novel agent must be demonstrated to offer benefits when used on top of existing standard-of-care therapy, which would include RAS blockade and quite probably today also SGLT2 inhibition. These practical limitations set aside, there is a growing interest in the importance of epigenetic processes in the development of diabetic kidney disease [116]. Given that several small molecule HDAC inhibitors suitable for in vivo administration have been developed, these tools could continue to be exploited to tease out the molecular causes of kidney disease in diabetes and this new knowledge may help to pave the way to novel treatment developments in the future.

## 11. Summary

In summary, despite improvements in clinical care, the evolving diabetes pandemic has ensured that kidney disease due to diabetes remains the most common cause of chronic kidney failure. In the search for new treatments, various repurposing opportunities have been explored, including the repurposing of HDAC inhibitors, initially developed for their use in hematological oncology. Over the past decade, a number of reports have emerged that describe a renoprotective effect of HDAC inhibitors in rodent models of diabetic kidney disease. In the non-diabetic setting, a comparable body of literature has reported potential anti-fibrotic renal effects of HDAC inhibition and HDAC inhibitors have also been reported to offer other metabolic benefits in diabetes. These findings have served as useful lenses through which the effects of epigenetic mechanisms in diabetes complications and kidney disease may be observed. Clinically approved HDAC inhibitors exhibit an adverse effect profile that would likely hinder their use for this indication. However, the development of better-tolerated agents and the discovery of coincident HDAC inhibitory effects of existing therapies (such as valproate) may offer an alternative route to the development of new treatments that reduce the occurrence of diabetic kidney disease through HDAC inhibition.

## Figures and Tables

**Table 1:** Pharmacological HDAC inhibitors, structural class, HDAC classes inhibited and indication or latest stage of development.

HDAC Inhibitor	Structural Class	HDAC Class Inhibited	Indication or Latest Phase of Development and Clinicaltrials.gov Identifier for that Condition
Vorinostat	Hydroxamic acid	Broad-spectrum	U.S. FDA approval (2006); cutaneous T cell lymphoma
Belinostat	Hydroxamic acid	Broad-spectrum	U.S. FDA approval (2014); peripheral T cell lymphoma
Panobinostat	Hydroxamic acid	Broad-spectrum	U.S. FDA approval (2015); multiple myeloma (in combination with bortezomib and dexamethasone)
Abexinostat	Hydroxamic acid	Broad-spectrum	Phase 1/2; sarcoma (NCT01027910)
Pracinostat	Hydroxamic acid	Broad-spectrum	Phase 3; acute myeloid leukemia (NCT03151408)
Resminostat	Hydroxamic acid	Broad-spectrum	Phase 2; advanced stage mycosis fungoides or Sézary syndrome (NCT02953301), Hodkin’s lymphoma (NCT01037478), hepatocellular carcinoma (NCT00943449)
Givinostat	Hydroxamic acid	Broad-spectrum	Phase 2/3; Duchenne muscular dystrophy (NCT03373968)
Quisinostat	Hydroxamic acid	Broad-spectrum	Phase 2; ovarian cancer (NCT02948075), cutaneous T cell lymphoma (NCT01486277)
Ricolinostat	Hydroxamic acid	HDAC6 (with some Class I inhibition)	Phase 2; diabetic neuropathic pain (NCT03176472)
Citarinostat	Hydroxamic acid	HDAC6 (with some Class I inhibition)	Phase 1; multiple myeloma (NCT02886065)
Dacinostat	Hydroxamic acid	Broad-spectrum	Not in clinical trial
Droxinostat	Hydroxamic acid	HDAC3, HDAC6, HDAC8	Not in clinical trial
Trichostatin A	Hydroxamic acid	Broad-spectrum	Not in clinical trial
Valproate	Short-chain fatty acid	I, II	U.S. FDA approval for seizures, bipolar disorder and migraine (more recently reported to have HDAC inhibitory effects)
Sodium butyrate	Short-chain fatty acid	I, II	Phase 2/3; schizophrenia (NCT02654405; NCT03010865)
Romidepsin	Cyclic peptide	I	U.S. FDA approval (2009); cutaneous T cell lymphoma and peripheral T cell lymphoma
Tacedinaline	Benzamide	I	Phase 3; lung cancer (NCT00005093)
Chidamide	Benzamide	I, IIb	China FDA approval (2014); peripheral T cell lymphoma
Mocetinostat	Benzamide	I, IV	Phase 2; urothelial carcinoma (NCT02236195), metastatic leiomyosarcoma (NCT02303262), non-small cell lung cancer (NCT02954991)
AR-42	Benzamide	I, II	Phase 1; renal cell carcinoma or soft tissue sarcoma (NCT02795819), vestibular schwannoma and meningioma (NCT02282917), acute myeloid leukemia (NCT01798901), multiple myeloma (NCT02569320), multiple myeloma, chronic lymphocytic leukemia or lymphoma (NCT01129193)
Entinostat	Benzamide	I	Phase 3; breast cancer (NCT03538171, NCT02115282)
Tubastatin A	Benzamide	HDAC6	Not in clinical trial
SK-7041	Hybrid, hydroxamic acid/benzamide	I	Not in clinical trial

**Table 2:** Articles reporting the effects of pharmacological HDAC inhibitors in experimental diabetic kidney disease.

Citation	HDAC Inhibitor Studied	HDAC Classes Inhibited	Experimental Models	Outcome
Noh et al., 2009 [54]	Trichostatin A SK-7041	Trichostatin A, Class I & II; SK-7041 Class I	STZ-diabetic rats, NRK-52E cells	Trichostatin A decreased proteinuria and extracellular matrix production; SK-7041 decreased matrix protein production in vitro
Gilbert et al., 2011 [6]	Vorinostat	Classes I & II	STZ-diabetic rats, NRK-52E cells	Vorinostat downregulated EGFR expression and decreased tubule cell proliferation and diabetes-associated kidney growth
Advani et al., 2011 [5]	Vorinostat	Classes I & II	STZ-diabetic wildtype and eNOS^−/−^ mice	Vorinostat downregulated eNOS and reduced oxidative stress, albuminuria and glomerular matrix production in STZ-diabetic wildtype mice but not STZ-diabetic eNOS^−/−^ mice
Khan et al., 2015 (1) [63]	Valproate	Classes I & II	STZ-diabetic rats	Valproate decreased tubule injury and renal fibrosis
Khan et al., 2015 (2) [64]	Valproate	Classes I & II	STZ-diabetic rats	Valproate decreased proteinuria and normalized NF-κB upregulation and autophagy downregulation
Sun et al., 2016 [65]	Valproate	Classes I & II	STZ-diabetic rats	Valproate decreased proteinuria, glomerular matrix deposition, endoplasmic reticulum stress and programmed cell death
Khan & Jena, 2014 [66]	Sodium butyrate	Classes I & II	STZ-diabetic rats	Sodium butyrate lowered plasma glucose and NF-κB expression and attenuated kidney injury and matrix deposition
Dong et al., 2017 [67]	Sodium butyrate	Classes I & II	STZ-diabetic wildtype and Nrf2^−/−^ mice	Sodium butyrate prevented Nrf2 downregulation and attenuated oxidative damage, inflammation, programmed cell death, fibrosis and albuminuria but was ineffective in STZ-Nrf2^−/−^ mice

eNOS = endothelial nitric oxide synthase; NF-κB = nuclear factor kappa-light-chain-enhancer of activated B cells; Nrf2 = nuclear factor erythroid 2-related factor 2.

**Table 3:** Common adverse effects and serious adverse effects of U.S. FDA approved HDAC inhibitors.

HDAC Inhibitor	Common Adverse Effects	Serious Adverse Effects
Belinostat	Nausea (42%), fatigue (37%), pyrexia (35%), anemia (32%), vomiting (29%), constipation (23%), diarrhea (23%), dyspnea (22%), rash (20%), peripheral edema (20%), cough (19%), thrombocytopenia (16%), pruritus (16%), chills (16%), decreased appetite (15%), abdominal pain (11%), hypotension (10%), phlebitis (10%), dizziness (10%)	Pneumonia, pyrexia, infection, anemia, increased creatinine, thrombocytopenia, and multi-organ failure (>2%)
Panobinostat (in combination with bortezomib and dexamethasone vs. placebo in combination with bortezomib and dexamethasone)	Arrhythmia (12%), diarrhea (68%), nausea (36%), vomiting (26%), fatigue (60%), peripheral edema (29%), pyrexia (26%), decreased weight (12%), decreased appetite (28%)	Pneumonia (18%), diarrhea (11%), thrombocytopenia (7%), fatigue (6%), sepsis (6%)
Romidepsin	Nausea (64%), diarrhea (36%), constipation (30%), hematological disorders (57%) including thrombocytopenia (41%), neutropenia (30%) and anemia (24%), asthenic conditions (55%), including fatigue (41%) and asthenia (16%), infections (55%), pyrexia (35%), anorexia (28%), dysgeusia (21%)	Infection (20%), pyrexia (8%), pneumonia, sepsis, vomiting (5%), cellulitis, deep vein thrombosis (4%), febrile neutropenia, gastrointestinal and abdominal pain (3%), chest pain, neutropenia, pulmonary embolism, dyspnea, dehydration (2%)
Valproate	Headache (31%), asthenia (27%), fever (6%), nausea (48%), vomiting (27%), abdominal pain (23%), diarrhea (13%), anorexia (12%), dyspepsia (8%), constipation (5), somnolence (27%), tremor (25%), dizziness (25%), diplopia (16%), amblyopia/blurred vision (12%), ataxia (8%), nystagmus (8%), emotional lability (6%), thinking abnormal (6%), amnesia (5%), flu syndrome (12%), infection (12%), bronchitis (5%), rhinitis (5%), alopecia (6%), weight loss (6%), depression (>5%), dyspnea (>5%), ecchymosis (>5%), increased appetite (>5%), insomnia (>5%), nervousness (>5%), peripheral edema (>5%), pharyngitis (>5%), thrombocytopenia (>5%), tinnitus (>5%), weight gain (>5%)	Hepatotoxicity, birth defects, pancreatitis, suicidal ideation, thromobocytopenia, hyperammonemia and hyperammonemic encephalopathy, hypothermia, multi-organ hypersensitivity reaction
Vorinostat	Fatigue (45%), diarrhea (47%), nausea (38%), dysgeusia (23%), thrombocytopenia (26%), anorexia (23%), decreased weight (20%), dry mouth (16%), vomiting (12%), increased blood creatinine (13%), alopecia (16%), decreased appetite (12%), muscle spasms (16%), anemia (13%), constipation (11%), chills (11%), dizziness (11%), abdominal pain (8%), proteinuria (8%), dyspnea (7%), headache (6%)	Pulmonary embolism (4.7%), anemia (2.3%)

Adapted from [117,118,119,120,121].
